# Is it time to focus on expiratory muscle training in children with asthma? A randomized controlled trial

**DOI:** 10.1007/s00431-026-07114-y

**Published:** 2026-06-03

**Authors:** Meltem Kaya, Hikmet Ucgun, Betul Gemici Karaaslan, Ayca Kiykim, Hilal Denizoglu Kulli

**Affiliations:** 1 Department of Physiotherapy and Rehabilitation, Faculty of Health Sciences, Istanbul Atlas University, Istanbul, Türkiye; 2 Department of Pediatric Allergy and Immunology, Cerrahpasa Faculty of Medicine, Istanbul University-Cerrahpasa, Istanbul, Türkiye

**Keywords:** Asthma, Expiratory muscle training, Functional capacity, Muscle strength, Pulmonary function

## Abstract

Respiratory muscle dysfunction contributes to reduced pulmonary and extrapulmonary outcomes in children with asthma. Although inspiratory muscle training has been widely studied, the effects of expiratory muscle training (EMT) in pediatric asthma remain unclear. This study investigated the effects of EMT on pulmonary function, respiratory and peripheral muscle strength, peak cough flow (PCF), functional capacity, and asthma control in children with asthma. This prospective, single-blinded randomized controlled trial included 30 clinically stable children with asthma aged 8–18 years. Participants were randomly assigned to an experimental group (EG) receiving EMT in addition to a home-based chest physiotherapy program or a sham group (SG) performing the same program with minimal resistance. EMT was performed once daily for 8 weeks at 30% of maximal expiratory pressure (MEP) with weekly load adjustments. Pulmonary function, respiratory and peripheral muscle strength, PCF, functional capacity, and asthma control test (ACT) scores were assessed at baseline and after 8 weeks. FVC and FEV₁ improved significantly only in the EG, whereas PEF increased in both groups with greater improvement in the EG. MIP increased in both groups, while MEP and MEP (% predicted) improved only in the EG. PCF, quadriceps strength, and 6MWT distance improved in both groups, with greater gains in the EG. ACT scores increased significantly in both groups but improved more in the EG.

*Conclusion*: Adding 8 weeks of EMT to chest physiotherapy improved pulmonary function, respiratory and peripheral muscle strength, cough effectiveness, functional capacity, and asthma control in children with asthma. EMT appears to be a safe and effective adjunct to pediatric asthma management.

*Trial registration*: The study was prospectively registered on the ClinicalTrials.gov website (registration number: NCT07169071; Date: 09/05/2025).

**Table:** 

**What is Known:** • *Respiratory muscle dysfunction is common in children with asthma and may contribute to reduced pulmonary function, ineffective cough, and decreased exercise capacity*. • *Most respiratory muscle training studies in asthma have focused primarily on inspiratory muscle training, while the role of expiratory muscle training remains largely unexplored*.	
**What is New:** • *This randomized controlled trial investigates the effects of expiratory muscle training (EMT) in children with asthma when combined with a home-based chest physiotherapy program*. • *The addition of 8 weeks of EMT resulted in greater improvements in pulmonary function, respiratory muscle strength, cough effectiveness, functional capacity, and asthma control compared with sham training*.

**What is Known:**

• *Respiratory muscle dysfunction is common in children with asthma and may contribute to reduced pulmonary function, ineffective cough, and decreased exercise capacity*.

• *Most respiratory muscle training studies in asthma have focused primarily on inspiratory muscle training, while the role of expiratory muscle training remains largely unexplored*.

**What is New:**

• *This randomized controlled trial investigates the effects of expiratory muscle training (EMT) in children with asthma when combined with a home-based chest physiotherapy program*.

• *The addition of 8 weeks of EMT resulted in greater improvements in pulmonary function, respiratory muscle strength, cough effectiveness, functional capacity, and asthma control compared with sham training*.

## Introduction

Asthma is one of the most common chronic respiratory diseases in childhood, affecting millions of children worldwide and imposing a significant burden on healthcare systems. It is characterized by chronic airway inflammation, reversible airflow obstruction, and bronchial hyperresponsiveness, leading to recurrent symptoms such as coughing, wheezing, and shortness of breath that vary in intensity and frequency [1]. These respiratory impairments can cause air trapping and inadequate alveolar ventilation, which increase breathing effort and exacerbate dyspnea, ultimately reducing exercise capacity and quality of life. Beyond airway pathology, asthma is also associated with extrapulmonary complications such as fatigue, peripheral muscle weakness, and decreased functional capacity [2].

Respiratory muscle dysfunction, observed even during stable phases, should be considered an important aspect in the long-term management of asthma [3]. Increased airway resistance and hyperinflation cause the diaphragm to flatten, placing it at a mechanical disadvantage and increasing the load on both inspiratory and expiratory muscles. Chronic mechanical stress, corticosteroid-induced myopathy, and inactivity further reduce maximal inspiratory (MIP) and expiratory pressures (MEP), leading to impaired ventilation, ineffective cough, and exercise intolerance in children with asthma [4].

Respiratory muscle training (RMT) has demonstrated significant benefits in various pulmonary and neurological disorders [5, 6], increasing respiratory muscle strength, enhancing exercise capacity, and improving quality of life. RMT can be categorized into two main modalities: inspiratory (IMT) and expiratory muscle training (EMT). EMT specifically targets the expiratory muscles, leading to increases in maximal expiratory pressure (MEP) and offering particular advantages in obstructive pulmonary diseases. Studies in conditions such as chronic obstructive pulmonary disease and cystic fibrosis have shown that EMT enhances cough effectiveness, airway clearance, and health-related quality of life [7, 8]. By improving expiratory muscle strength and ventilatory efficiency, EMT may also positively influence speech, swallowing, and overall physical performance, supporting its potential relevance in pediatric asthma [9]. Despite this growing body of evidence, most studies in asthma have primarily focused on IMT, which effectively improves MIP and reduces exertional dyspnea, particularly when performed at high intensity and for extended durations [4]. Although IMT has demonstrated benefits for both pulmonary and extrapulmonary outcomes, the role of EMT remains largely unexplored in pediatric asthma. Therefore, the present study aimed to investigate the effects of EMT on pulmonary function, respiratory and peripheral muscle strength, peak cough flow (PCF), functional capacity, and asthma control in children with asthma.

## Methods

### Study design and subjects

This prospective, single-blinded randomized controlled trial was conducted between May 2024 and July 2025. Children with mild-to-moderate asthma aged 8–18 years were recruited from the Department of Pediatric Allergy and Immunology of a university hospital for respiratory physiotherapy. Inclusion criteria were a confirmed diagnosis of asthma, established by pediatric allergy and immunology specialists according to international guideline criteria, clinical stability, ability to cooperate, and willingness to participate. Patients were excluded if they had a recent upper respiratory infection, asthma exacerbation, medication change within the previous three weeks, hospitalization, transplantation, musculoskeletal deformities affecting respiratory function, or < 80% adherence to the exercise program. Participants were randomly assigned to the experimental group (EG) or sham group (SG) using a computer-generated sequence. A blinded researcher performed outcome assessments, while another researcher supervised the interventions. Evaluations were repeated after 8 weeks. Participants kept an exercise and physical activity diary monitored weekly by telephone, and adherence was calculated as completed sessions divided by total sessions.

The study was approved by the Ethics Committee of Istanbul Atlas University (No: 10/15) and registered at ClinicalTrials.gov (NCT07169071). Written informed consent was obtained from parents or legal guardians.

### Outcome measures

Primary and secondary outcomes were predefined. The primary outcomes were MIP and MEP. Secondary outcomes included pulmonary function parameters, PCF, peripheral muscle strength, functional capacity, and asthma control.

Demographic and clinical characteristics of patients, including gender, age, height, weight, age at diagnosis, presence of chronic diseases, medications, asthma attacks in the previous year, and number of hospitalizations, were collected.

### Pulmonary function

Spirometry measurements were performed in the pre-bronchodilator (pre-BD) state using a spirometer (COSMED Pony FX, COSMED, Rome, Italy), in accordance with the American Thoracic Society (ATS) and European Respiratory Society (ERS) guidelines to ensure standardized assessment of pulmonary function [10]. The measured parameters, including forced vital capacity (FVC), forced expiratory volume in one second (FEV_1_), FEV_1_/FVC ratio, and peak expiratory flow (PEF), were reported as percentages of the predicted values.

### Respiratory muscle strength

MIP and MEP were measured using an electronic mobile device (MicroRPM, Micro Medical; UK). Participants were familiarized with the MIP and MEP procedures through standardized instructions and practice trials before data collection to minimize potential learning effects. Participants performed at least three maximal inspiratory and expiratory maneuvers, and additional attempts were permitted until two reproducible measurements within 5% of each other were achieved. The highest value was used for analysis [11].

### Peak cough flow

PCF was measured using a portable peak flow meter (ExpiRite Peak Flow Meter, China). Participants were instructed to perform a maximal cough following full inspiration while sitting and wearing a nose clip. At least three trials were conducted, and the highest value was recorded for analysis. Standardized verbal encouragement was provided to ensure maximal and reproducible effort [12].

### Peripheral muscle strength

Quadriceps muscle strength test was performed using a MicroFet2 hand-held dynamometer (Hogan Health Industries Inc.) following the break method [13]. The maximum value of three consecutive measurements taken from the dominant side was recorded. Children were given approximately 1 min of rest between efforts.

### Functional capacity

Functional capacity was assessed using the six-minute walk test (6MWT) according to ATS guidelines along a 30-m indoor corridor. Participants were instructed to walk for 6 min at a self-selected brisk pace without running. Oxygen saturation, heart rate, respiratory rate, and blood pressure were recorded before and after the test, while dyspnea, fatigue, and leg pain were documented. The total distance walked was recorded in meters [14].

### Asthma control

The asthma control test (ACT) assesses a patient’s asthma control over the past 4 weeks, covering activity limitations and symptoms. Scores range from 1 (poor) to 5 (best), with a maximum of 25. Asthma is well-controlled if the score is > 20, partially controlled if 16–19, and uncontrolled if ≤ 15 [15].

### Interventions

Participants in both groups were instructed to perform a home-based chest physiotherapy program consisting of diaphragmatic breathing, thoracic expansion exercises, breathing control techniques, cough training, and relaxation positions. The program was prescribed for 30 min per day, 5 days per week, for 8 weeks. The first session was supervised by a physiotherapist to ensure correct technique, and participants received a written brochure to support independent home practice. In addition to this structured intervention, participants were advised to engage in at least 60 min of moderate-intensity physical activity daily, defined as activities that increase breathing and heart rate while still allowing comfortable conversation [16].

In addition to this program, participants in the EG performed EMT using the POWERbreathe EX1 Medic device (POWERbreathe International Ltd., Southam, Warwickshire, UK) once daily for 25 breaths (1-min rest after every five breaths) at 30% of MEP for 8 weeks. The intervention duration of 8 weeks was selected based on previous respiratory muscle training studies indicating that this period is sufficient to induce meaningful physiological adaptations; resistance was adjusted weekly according to updated MEP values to ensure progressive overload [17]. The SG followed the same protocol but performed the exercises with minimal resistance (approximately 10 cmH₂O), corresponding to the lowest resistance level recommended by the manufacturer.

Safety was monitored throughout the study by supervising physiotherapists. Participants and their parents were instructed to report any asthma-related symptoms, such as wheezing, chest tightness, or shortness of breath. Usual asthma medications were maintained during the study period, and rescue bronchodilators were available if needed.

### Statistical analysis and sample size

SPSS v.26 (SPSS Inc., USA) was used for data analysis. The normality of data distribution was tested with the Shapiro–Wilk test. Chi-square tests were used to compare categorical variables between groups. Depending on data distribution, paired samples *t*-test or independent samples *t*-test was used for within-group comparisons, and Wilcoxon or Mann–Whitney *U* tests were used for between-group comparisons. Statistical significance was set at *p* < 0.05. Effect size (Cohen’s d) was calculated, with values of 0.2, 0.5, and 0.8 indicating small, moderate, and large effects, respectively [18].

G*Power v3.1 software (Universität Kiel, Germany) was used to determine the sample size [19]. G*Power v3.1 software (Universität Kiel, Germany) was used to determine the sample size [17]. MEP was selected as the reference parameter. The calculation was based on a previous study evaluating EMT in children with cystic fibrosis [7], which reported an effect size (Cohen’s d) of 1.496. Based on this effect size, a minimum of 13 participants per group was required. Considering a possible 20% attrition rate during the 8-week intervention, the sample size was increased to 15 participants per group.

## Results

**Fig. 1:**
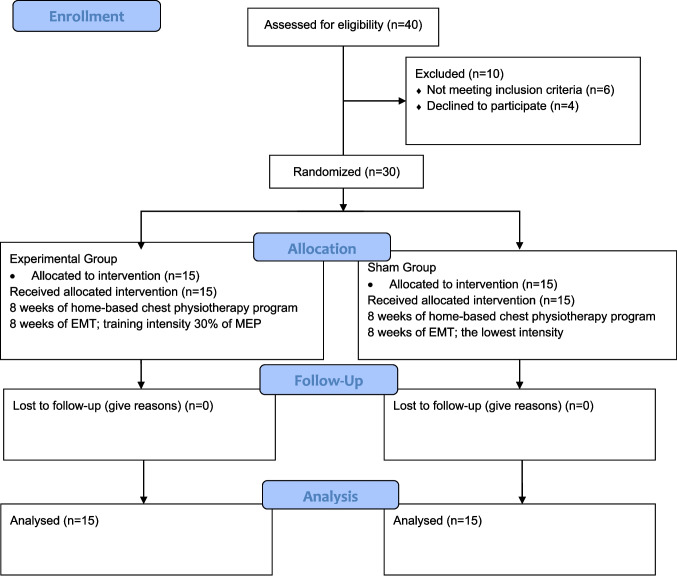
Flow chart of the study

Forty children with asthma were assessed for eligibility. Thirty children who met the inclusion criteria were included and randomized, and all completed the study without any dropouts or exclusions related to poor adherence (< 80%) (Fig. 1). No adverse events related to the EMT intervention were reported during the 8-week study period. The demographic and clinical characteristics of the participants are presented in Table 1, with no significant differences between groups. Adherence to the exercise program was 88.88 ± 4.31% in the EG and 83.30 ± 1.41% in the SG (*p* = 0.296). Compliance with physical activity recommendations was also similar between groups (EG, 80.95 ± 7.20%; SG, 79.24 ± 6.45%; *p* = 0.858). Intergroup and intragroup comparisons are shown in Table 2. FVC% and FEV₁% improved significantly only in the EG (*p* = 0.018 and *p* = 0.025, respectively), whereas PEF% increased significantly in both groups (EG, *p* = 0.028; SG, *p* = 0.048), with a greater improvement in the EG (*p* = 0.021). No significant change was observed in FEV₁/FVC%. MIP increased in both groups but was greater in the EG (*p* = 0.045, *d* = 0.60), whereas MEP improved significantly only in the EG, with a significant between-group difference (*p* = 0.012, *d* = 0.75). MIP (%pred) increased in both groups, while MEP (%pred) improved only in the EG, favoring the EG in the between-group comparison (*p* = 0.020). PCF, quadriceps muscle strength, and 6MWT distance improved in both groups, with greater improvements in the EG (*p* = 0.018, *p* = 0.034, and *p* = 0.026, respectively). ACT scores also increased in both groups, but improved significantly more in the EG (*p* = 0.008, *d* = 0.82).

**Table 1:** Demographic and clinical characteristics of the participants

	EG (*n* = 15)	SG (*n* = 15)	*p*-value
Age (years)	12.21 ± 2.93	13.17 ± 2.26	0.454
Gender Female Male	6 (40%) 9 (60%)	6 (40%) 9 (60%)	1.000
Body composition			
Weight (kg)	50.15 ± 14.55	49.30 ± 15.06	0.801
Height (cm)	152.84 ± 12.91	154.08 ± 14.20	0.728
BMI (kg/m^2^)	21.30 ± 4.45	20.67 ± 3.48	0.810
Duration of diagnosis (months)	58.46 ± 46.95	57.07 ± 49.94	0.669
Drugs, number of users, n (%)			
Inhaled corticosteroids	9 (60%)	8 (53.3%)	0.616
β2 agonists	12 (80%)	13 (86.6%)	
Number of exacerbations in the past year	0.53 ± 0.87	0.76 ± 0.97	0.463
Presence of chronic disease			
Yes	14 (93.3%)	15 (100%)	0.710
No	1 (6.6%)	0	
ACT			
Controlled	3 (20%)	5 (33.3%)	0.345
Partially controlled	7 (46.7%)	6 (40%)	
Uncontrolled	5 (33.3%)	4 (26.7%)	
Adherence to exercise program (%)	88.88 ± 4.31	83.30 ± 1.41	0.296
Compliance with physical activity recommendation (%)	80.95 ± 7.20	79.24 ± 6.45	0.858

Data are presented as mean ± standard deviation or n (%). *ACT* asthma control test, *BMI* body mass index, *ICS* inhaled corticosteroids

**Table 2:** Effects of EMT and conventional chest physiotherapy on pulmonary function, respiratory and peripheral muscle strength, functional capacity, PCF, and asthma control

	EG (*n* = 15)			SG (*n* = 15)				
	Baseline	Post-training	Intragroup differences*	Baseline	Post-training	Intragroup differences*	Intergroup differences*	Effect size (Cohen’s d)
Pulmonary function								
FVC (% pred)	94.47 ± 12.44	98.29 ± 7.95	*p* = 0.018*	92.47 ± 11.44	90.70 ± 5.69	*p* = 0.613	*p* = 0.012*	0.71
FEV_1_ (% pred)	84.15 ± 9.22	90.29 ± 11.07	*p* = 0.025*	82.82 ± 16.22	83.00 ± 5.39	*p* = 0.748	*p* = 0.038*	0.51
FEV_1_/FVC (% pred)	79.73 ± 11.84	81.34 ± 10.35	*p* = 0.734	77.73 ± 13.84	83.11 ± 8.12	*p* = 0.545	*p* = 0.408	0.13
PEF (% pred)	76.08 ± 13.85	84.17 ± 16.42	*p* = 0.028*	74.82 ± 16.49	79.47 ± 12.52	*p* = 0.048*	*p* = 0.021*	0.61
Respiratory muscle strength								
MIP (cm H_2_O)	59.76 ± 28.56	89.72 ± 15.56	*p* = 0.006*	53.30 ± 34.84	67 ± 19.93	*p* = 0.035*	*p* = 0.045*	0.60
MIP (% pred)	69.82 ± 12.22	80.64 ± 10.99	*p* = 0.025*	65.43 ± 25.46	72.10 ± 8.76	*P* = 0.040*	*p* = 0.020*	0.68
MEP (cm H_2_O)	55.46 ± 27.57	78.12 ± 23.11	*p* = 0.005*	59.76 ± 28.56	68.80 ± 20.14	*p* = 0.192	*p* = 0.012*	0.75
MEP (% pred)	51.00 ± 13.62	68.52 ± 10.62	*p* < 0.001*	54.29 ± 8.49	59.27 ± 11.23	*p* = 0.070	*p* = 0.020*	0.70
Peak cough flow (L/min)	368.12 ± 88.87	433.75 ± 103.69	*p* < 0.001*	345.70 ± 74.25	375.15 ± 85.50	*p* < 0.001*	*p* = 0.018*	0.81
Peripheral muscle strength								
Quadriceps strength (kg)	33.72 ± 7.37	37.55 ± 9.00	*p* < 0.001*	29.61 ± 7.03	30.18 ± 7.01	*p* < 0.001*	*p* = 0.034*	0.58
Functional capacity								
6MWT distance (m)	552.23 ± 68.12	590.14 ± 55.23	*p* = 0.035*	557.13 ± 49.29	577.14 ± 51.23	*p* = 0.045	*p* = 0.026*	0.64
Asthma control (ACT)	17.16 ± 3.95	21.75 ± 3.07	*p* = 0.002*	18.83 ± 4.58	20.58 ± 4.58	*p* = 0.036*	*p* = 0.008*	0.82

Data are presented as mean ± standard deviation

*ACT* asthma control test, *FEV₁* forced expiratory volume in one second, *FVC* forced vital capacity, *MIP* maximal inspiratory pressure, *MEP* maximal expiratory pressure, *PEF* peak expiratory flow, *6MWT* six-minute walk test. *p < 0.05

## Discussion

This study investigated the effects of EMT on pulmonary function, respiratory and peripheral muscle strength, PCF, functional capacity, and asthma control in children with asthma. To our knowledge, this is the first randomized controlled trial evaluating the isolated impact of EMT in pediatric asthma. The findings showed that adding EMT to standard chest physiotherapy resulted in greater improvements in pulmonary function, respiratory and peripheral muscle strength, cough effectiveness, functional capacity, and asthma control compared with the SG.

Pulmonary function outcomes observed in the present study provide important insights into the respiratory adaptations induced by EMT in pediatric asthma. The integration of EMT into a chest physiotherapy program led to greater improvements in FVC%, FEV₁%, and PEF% compared with conventional methods alone, highlighting the clinically meaningful contribution of expiratory muscle performance to ventilatory efficiency. While asthma management traditionally prioritizes pharmacological control of airway inflammation, our findings emphasize the importance of respiratory muscle mechanics in compensating for the mechanical constraints imposed by airflow limitation. In pediatric asthma, airway obstruction and air trapping can place respiratory muscles at a mechanical disadvantage; strengthening expiratory muscles may therefore facilitate lung emptying and improve expiratory flow [20]. This mechanistic interpretation aligns with the findings of Elnaggar et al. [21], who demonstrated significant spirometric gains when the expiratory phase was specifically targeted in children with asthma. In contrast, the meta-analysis by Lista-Paz et al. [4] reported that although respiratory muscle training primarily IMT consistently improves muscle strength, its effects on resting spirometric indices such as FEV₁ and FVC are often inconsistent, suggesting that interventions specifically directed at expiratory loading may exert a more direct influence on flow-dependent measures. It should also be noted that both groups in the present study received a structured home-based chest physiotherapy program including breathing exercises. Breathing-based interventions have been shown to improve pulmonary function test parameters in children with asthma, likely by facilitating prolonged and more effective expiratory maneuvers and improving ventilatory control [22]. The improvement in PEF observed in both groups may therefore be partly attributed to repeated expiratory practice and high adherence to the 8-week program. However, the greater magnitude of change in the EMT group suggests that externally loaded, repetitive expiratory resistance provides an additional training stimulus beyond breathing exercises alone. Thus, the greater PEF response in the EMT group likely reflects enhanced expiratory muscle performance, with potential implications for airway clearance and clinical stability. No significant change was observed in FEV₁/FVC%. The absence of a significant change in the FEV₁/FVC ratio may be attributed to the relatively preserved baseline pulmonary function of the participants, most of whom had mild-to-moderate asthma, as well as to the proportional improvements in both FEV₁ and FVC, resulting in minimal alteration of this ratio.

In the present study, MIP increased in both groups, whereas MEP improved exclusively in the EG, with a greater increase in MIP observed following EMT. The finding is clinically relevant, as respiratory muscle dysfunction has been reported even in clinically stable children with asthma due to chronic airflow limitation, increased work of breathing, dynamic hyperinflation, and factors such as corticosteroid exposure or reduced physical activity [23]. The improvement in MIP observed in both groups may be partly attributed to repetitive inspiratory activation during structured breathing exercises included in the home-based chest physiotherapy program. Supporting this interpretation, an 8-week balloon-breathing exercise program has been shown to significantly increase both MIP and MEP in children with asthma [24], and Nield et al. [25] demonstrated that pursed-lip breathing enhances MIP in patients with chronic obstructive pulmonary disease by inducing repetitive and forceful expiratory efforts. The selective improvement in MEP in the EG likely reflects the task-specific overload provided by progressive expiratory resistance, enhancing motor unit recruitment and pressure-generating capacity of the abdominal and internal intercostal muscles. Despite the expiratory focus of the intervention, the increase in MIP may indicate a synergistic cross-training effect. Strengthening expiratory muscles may facilitate more complete exhalation, reduce air trapping, and improve diaphragm positioning, thereby enhancing inspiratory efficiency. Consistent with the ideas of Elnaggar et al. [21], targeting the expiratory phase may reduce mechanical load across the respiratory cycle and indirectly support inspiratory performance. Another possible mechanism linking respiratory muscle strengthening to the observed improvement in pulmonary function test is the enhanced mechanical contribution of the respiratory muscles to thoracic expansion. Increased pressure-generating capacity may improve ventilatory efficiency and reduce the energy cost of breathing, thereby contributing to higher FEV₁ and FVC values [26]. Thus, the improvements observed in the EG further support the functional relevance of the increases in MIP and MEP. The moderate-to-large effect sizes observed for MEP and MIP further suggest that EMT provides a more potent stimulus for neuromuscular adaptation than conventional chest physiotherapy alone. While systematic reviews have predominantly emphasized IMT [4], our findings highlight the expiratory component as a crucial yet often underrecognized target in pediatric asthma management.

The current study demonstrated a significant and greater increase in PCF in the EG compared to the SG. Effective cough generation depends on both expiratory muscle strength and the inspiratory lung volume achieved before the expulsive phase [27]; therefore, the observed improvement in PCF likely reflects the combined influence of enhanced MEP together with gains in MIP and FVC%. Strengthening of the abdominal and internal intercostal muscles may have increased intrathoracic pressure during forced expiration, thereby augmenting airflow velocity, while improved inspiratory capacity may have optimized the volume-dependent component of cough performance. In addition, reductions in air trapping and improved thoracoabdominal coordination may have further contributed to more efficient cough mechanics. Similar improvements in cough performance following EMT have been documented across diverse clinical populations, including patients with stroke and Parkinson’s disease [28, 29], as well as in individuals with neuromuscular and chronic lung diseases such as cystic fibrosis [7, 30]. The parallel improvements in MEP and PCF observed in the present study reinforce the task-specific nature of EMT, suggesting that progressive expiratory resistance provides a sufficient overload stimulus to translate isolated strength gains into measurable functional outcomes. Moreover, the improvement in ACT further suggests that enhanced cough efficiency may support better secretion clearance and contribute to greater clinical stability.

Quadriceps muscle strength increased significantly in both groups, with greater improvement in the EG. This improvement in both groups may be attributed to the structured home-based physiotherapy program and high adherence to daily physical activity recommendations. Regular participation in moderate-intensity activity and breathing exercises may have reduced dyspnea and encouraged greater daily movement, indirectly promoting peripheral muscle adaptation. However, the larger improvement in the EG suggests that EMT may provide an additional physiological stimulus beyond general physical activity. To our knowledge, no previous study has specifically examined the effects of isolated EMT on peripheral muscle strength in children with asthma. A plausible mechanism underlying this greater improvement involves modulation of the respiratory muscle metaboreflex [31]. In chronic respiratory conditions, respiratory muscle fatigue can trigger sympathetic vasoconstriction in peripheral limbs to prioritize blood flow to the diaphragm. By strengthening expiratory muscles and reducing the work of breathing, EMT may delay this reflex, helping preserve peripheral muscle perfusion and improve exercise performance [32]. Evidence from other chronic disease populations, including COPD and heart failure, indicates that respiratory muscle training can improve peripheral muscle oxygenation and functional capacity [33, 34], supporting the concept of a systemic rather than purely localized effect. In line with this perspective, gamification-based breathing interventions in children with asthma have also been associated with improvements in extrapulmonary outcomes in addition to pulmonary benefits, supporting the concept that enhancing respiratory mechanics may positively influence overall physical performance [35]. Taken together, these findings suggest that although structured physiotherapy and increased physical activity provide a fundamental stimulus for peripheral muscle adaptation, the addition of EMT may further amplify these effects by optimizing ventilatory efficiency, lowering the metabolic cost of breathing, and enabling greater peripheral muscle engagement during daily activities.

Functional capacity, assessed by the 6MWT, improved significantly in both groups; however, the magnitude of improvement was significantly greater in the EG. Importantly, the mean increase observed in the EG exceeded the minimal clinically important difference reported for pulmonary diseases [36], indicating that the improvement was not only statistically significant but also clinically meaningful. Given that respiratory muscle strength—particularly MIP and MEP—has been identified as an important determinant of functional exercise capacity in children with asthma [3], the greater improvement in walking distance in the EG may be attributed to the concomitant improvements in respiratory muscle function. Another possible explanation for the enhanced functional capacity may be improved ventilatory efficiency and reduced relative respiratory load during submaximal exertion. In children with asthma, exercise tolerance can be limited by increased work of breathing and early respiratory muscle fatigue [37]. By strengthening the expiratory muscles, EMT likely improves expiratory flow generation and overall ventilatory mechanics, thereby decreasing the proportion of total effort devoted to breathing during exercise. This reduction in ventilatory demand may delay respiratory muscle fatigue and allow a greater contribution from peripheral musculature, ultimately improving functional capacity. These findings align with previous evidence suggesting that respiratory muscle training can enhance exercise capacity when applied with adequate intensity and duration. Consistent with our results, Emirza et al. reported significant improvements in 6MWD accompanied by increases in MIP and MEP following EMT in children with cystic fibrosis [7]. Taken together, these findings suggest that EMT may improve functional capacity by enhancing ventilatory efficiency and supporting more effective respiratory–peripheral muscle coordination during exercise. These adaptations may enable children with asthma to tolerate higher levels of physical activity and contribute positively to long-term functional outcomes.

Asthma control, as assessed by the ACT, improved significantly in the EG, indicating that the physiological enhancements achieved through EMT were reflected in improved symptom control and disease management. Asthma control is a multidimensional construct that encompasses symptom frequency, activity limitation, rescue medication use, and overall perception of disease stability. Therefore, improvements in respiratory muscle performance, particularly MIP and MEP, may influence ACT outcomes by reducing exertional dyspnea and improving tolerance to daily activities. As respiratory muscles become stronger and more efficient, the relative ventilatory load during routine physical tasks decreases, which may attenuate symptom perception and contribute to improved self-reported control. The present findings seem to be consistent with previous research showing that combined RMT improves ACT scores in children with asthma [21]. The authors stated that targeting both phases of breathing enhances asthma control, likely through improvements in respiratory muscle strength and pulmonary function [21]. Similarly, Bunlam et al. reported that expiration-based balloon-blowing breathing exercises performed for 8 weeks significantly increased both MIP and MEP and were accompanied by significant improvements in asthma control scores in school-age asthmatic children [24]. Although the intervention modalities differed, both studies support the concept that strengthening respiratory musculature can translate into clinically relevant improvements in asthma symptom management. Beyond direct muscular effects, breathing interventions may enhance asthma control through behavioral and self-management mechanisms. Structured respiratory training may improve breathing awareness and pattern regulation, reducing anxiety related to dyspnea. In children, consistent home-based programs supported by parental involvement can strengthen adherence and promote active symptom management. This perspective aligns with GINA recommendations emphasizing self-management and family engagement in asthma care [38].

Several limitations should be acknowledged. Only clinically stable children with mild-to-moderate asthma were included, which may limit generalizability to patients with more severe diseases. In addition, although adherence to the training program and physical activity recommendations was monitored, daily physical activity was not objectively measured. Thirdly, the relatively short intervention period prevents conclusions about the long-term sustainability of the observed improvements. Finally, although including participants aged 8–18 years enhances the generalizability of the findings, this broad age range may introduce heterogeneity related to growth and pubertal development. Additionally, MIP, MEP, and PCF are effort-dependent measures; therefore, the observed improvements may have been influenced in part by participants’ familiarization with the testing maneuvers and techniques, despite the standardized practice trials conducted before assessment.

## Conclusion

In conclusion, adding 8 weeks of progressive EMT to a standard chest physiotherapy program resulted in greater improvements in pulmonary function, respiratory and peripheral muscle strength, cough effectiveness, functional capacity, and asthma control in children with asthma. These findings suggest that EMT is a safe, low-cost, and effective non-pharmacological adjunct to pediatric asthma management. Future studies should investigate the long-term sustainability of these benefits and the effectiveness of EMT across different asthma phenotypes and severity levels.

## Data Availability

No datasets were generated or analysed during the current study.

## Abbreviations

6MWT: Six-minute walk test
ACT: Asthma control test
ATS: American thoracic society
EG: Experimental group
EMT: Expiratory muscle training
ERS: European respiratory society
FEV₁: Forced expiratory volume in one second
FVC: Forced vital capacity
IMT: Inspiratory muscle training
MEP: Maximal expiratory pressure
MIP: Maximal inspiratory pressure
PCF: Peak cough flow
PEF: Peak expiratory flow
RMT: Respiratory muscle training
SG: Sham group
